# Retailing Strategies for Power Retailers with a Generator Background Considering Energy Conservation Services under the Internet of Things

**DOI:** 10.3390/s22176612

**Published:** 2022-09-01

**Authors:** Xun Dou, Jiazhe Zhou, Yanbo Ding, Jiacheng Li, Yang Cao, Maohua Shan, Hao Yuan

**Affiliations:** 1 College of Electrical Engineering and Control Science, Nanjing Tech University, Nanjing 211816, China; 2 China Electric Power Research Institute (Nanjing), Nanjing 210003, China

**Keywords:** smart grid, internet of things technology, energy conservation, power retailers with generator background, system dynamics, retail strategy

## Abstract

Facing the electricity market environment, in which the traditional power grid is transformed into a smart grid, power retailers with a generator background are designing new business models of cold-heat-electricity multi-energy supply based on the Internet of Things data collection, interconnection, computing and other technical supports. On the other hand, through internet of things real-time monitoring technology, the necessity of setting up energy security for power retailers is explored to enhance the control’s ability to deal with the risks of electricity sales. Firstly, based on internet of things data analysis, retail strategies such as cooling-heat-electricity multi-energy packages, desulphurization and carbon emissions and energy conservation are designed. Then, a revenue cost measurement model based on the generator background of the power retailers is established. A source of data for the expansion of power retailers and the proliferation of load users is provided through the real-time monitoring of new business models that consider the operation of energy conservation on the supply and use side. Finally, an analysis based on the detection of operation under the scenarios constructed in the example of coal price market fluctuations and proliferation stagnation of user-side packages is conducted. It is verified that the power retailers with a generator background can effectively weaken the adverse impact of upward fluctuations in the coal price market in the peak season of energy consumption on the cost of power retailers by setting energy conservation. At the same time, the diffusion of a new business model in the user side is improved, and the revenue source of power retailers is further expanded. Therefore, taking energy conservation as an important innovation technique of retail strategy can enhance the market competitiveness and risk control ability of power retailers.

## 1. Introduction

As the smart grid continues to develop, Internet of Things (IOT) technology is playing an important role in the construction of the electricity market and is particularly instructive for the progress of power retailers in terms of sensors, communications and cloud computing. However, the characteristics and operating mechanism of the current new power system are becoming increasingly complex, and the construction of the power market faces multiple objectives such as ensuring supply, promoting transformation, improving efficiency and optimizing resource allocation [1], as well as the development of reasonable supportive and regulating green and low-carbon advanced coal power [2]. In this context, under the requirements of a smart grid with low carbon, environmental protection and efficient supply assurance, how power retailers can give full play to the advantages of IOT technology and their own background as generators to participate in the power sales market and meet customers’ needs has become a hot topic of current research. In the current situation of tight supply and demand balance, power retailers have detected that their costs are highly susceptible to growth due to the upward trend of the coal price market through data collection, interconnection, calculation and other IOT technologies, which has resulted in a lack of momentum in the development of power retailers. As a result, high supply prices are set to sustain the expansion of the power retailers, shifting the cost pressure to the load users and slowing the diffusion of the new business model on the customer side. In this regard, it is necessary to carry out data collection, calculation, monitoring and other IOT technology support work for the electricity sales market with power retailers with a generator background. Moreover, based on data from the overall electricity sales environment, a new multi-energy retail package for power retailers is designed. The significance of the energy assurance service in reducing the cost of energy supply and ensuring a strong supply in the context of the smart grid is important for power retailers to develop retail strategies that take energy assurance into account.

In terms of retailing strategies for power retailers in the context of generators, current research has focused on innovative marketing strategies for electricity sales. In [3], strengths, weaknesses, opportunities and threats (SWOT) analysis was used to propose marketing strategies for a variety of power retailers with generator background from traditional, portfolio and internet. In response to the overall development strategy objectives of the new round of national electricity system reform [4], generators have been actively developing marketing strategies for power sales including offering integration, industry chain extension and promoting optimal resource allocation [5] to expand their business development space and improve overall efficiency. Based on the fact that generators had mostly provided direct supply business to large customers in the past, power retailers with the same background should continue to consolidate and develop, as proposed in [6]. However, the need for power retailers to accumulate experience in technology for new energy services, energy saving, and other businesses was proposed in [7]. In addition, the characteristics of coal-fired cogeneration systems were combined by power retailers with a coal-fired power producer background to take full account of the increasing demand for residential heating. A retail strategy for waste heat supply at the cold end of combined heat and power (CHP) systems was proposed to address the problems of insufficient peaking capacity and insufficient heat supply to the system [8,9,10]. At the same time, in the context of decentralized decision making in the IOT [11], a variety of optimized operation models for power sales companies are proposed with the goal of maximizing the benefits for both power sales companies and all customers [12]. However, in the current study, many power retailers with a generator background monitor the cost of electricity sales and strengthening energy saving and consumption reduction according to their own strengths and areas. Additionally, tailoring retail solutions based on customer characteristics is also the development path for power retailers in the new situation. The multi-energy supply to users by power retailers with a generator background is often expensive, lacking more reasonable electricity prices and richer alternative energy retail strategies. In addition, it is also an urgent social responsibility and obligation for power retailers to actively build a low-carbon desulfurization environmental protection industry chain.

In terms of energy conservation services for power retailers, many of them had adopted measures to cope with the risks faced in the purchase and sale of electricity under the new competitive market segment, adapting to a variety of market risks. Power retailers were exposed to multiple time scales and spatial scales of power purchase in electricity market transactions [13]. With the objective of maximizing the comprehensive utility of expected profit and conditional risk loss [14], a variety of risk-averse strategies for power retailers including power purchase portfolios [15] and power sales pricing was established. Additionally, in this type of strategy, the market-based insurance mechanism for electricity price fluctuations is taken into account in the context of the IOT [16]. However, current research had been based on the risks associated with the purchase and sale of electricity by the power retailers themselves. Real-time monitoring and control of customer-side risks were overlooked, including the possibility of load customers choosing other power retailers due to “unavailability” or “high costs”. In turn, the lack of momentum in the development of power retailers, as well as the potential bankruptcy risk due to the upward fluctuation of coal price.

To address the above issues, based on the existing research, a retail strategy model for power retailers with a generator background considering energy conservation services is established through IOT technology and the integration of real-time monitoring data of energy demand of load users in the region. By analyzing the demand for energy assurance services for power retailers, a retail strategy for the development of energy assurance services by power retailers in a competitive electricity sales market environment is designed. Slow proliferation or even recession due to compromised customer interests is effectively avoided, the competitiveness of generators is enhanced, and the smooth operation and healthy development of the electricity retail market is maintained.

The main innovations of this paper are as follows:

- Based on the collection, monitoring and calculation of environmental data in the electricity sales market based on IOT technology, the real-time monitoring data of energy demand of load users are used as an important basis for CHP and waste heat refrigeration supply energy for power retailers with a generator background. The new business model of cooling-heating-electricity multi-energy packages is innovatively proposed, while data analysis of costs, revenues, expansion scale of power retailers and the number of proliferations of new packages is conducted to verify the necessity of setting up energy guarantee services.
- With energy assurance services being set up by power retailers with a generator background, the proportion of coal reserves and energy losses in the cost of coal purchased by power retailers is reduced, the adverse impact of upward market fluctuations in coal prices on the costs of power retailers during the peak energy season is weakened and the expansion rate of power retailers in the market is released.
- By setting up an energy conservation service, the power retailers compress the cost of energy sales, thus reducing the cost for the load customers by making heat and cold energy a complimentary part of the electricity supply in the new package and not charging for it separately. This is conducive to safeguarding the energy rights of load customers and increasing the proliferation of the new business model on the customer side, as well as further expanding the revenue stream of the power retailers and improving its risk management capabilities.

The organizational structure of this paper is as follows. The retail strategy model for power retailers considering energy conservation services is built in Section 2, and the modeling process is clarified. In Section 3, the retailing strategies for power retailers based on the background of generators is established, as well as mathematical and strategic model for power retailers to promote new business models is provided. In Section 4, the effectiveness of the energy assurance service in scenarios of coal price market volatility and customer-side diffusion stagnation is verified by means of an arithmetic test analysis.

## 2. Retail Strategy Model for Power Retailers Considering Energy Conservation Services

### 2.1. Modeling Principles

**Figure 1:**
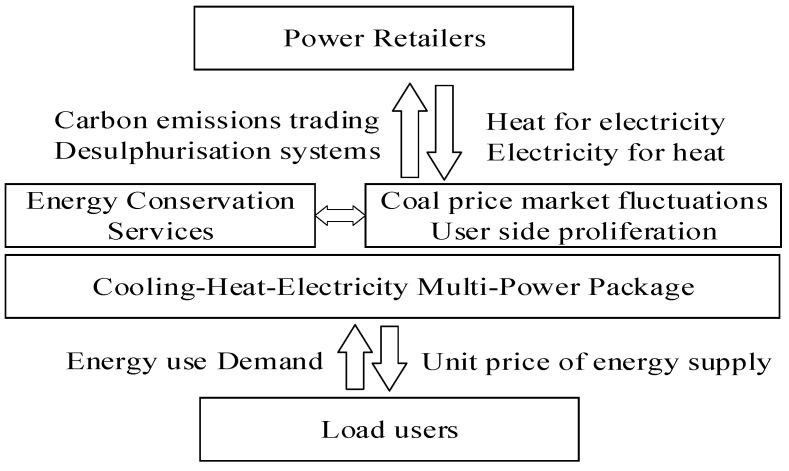
Schematic diagram of a cold-heat-electricity multi-energy package considering energy conservation services.

The energy demand of the load users at different times is collected and monitored in real time, and power retailers with a generator background determine the amount of cold energy, heat energy and electricity to be supplied to the load users by using heat to determine electricity or electricity to determine heat, respectively. While the unit price of energy supply is set at different time periods to generate revenue from the sale of energy, based on the measured emissions of sulfur dioxide and carbon dioxide from coal combustion by the electricity producer, the power retailers pay for the operation of the desulphurization system and the cost of participating in the carbon trading market. In order to cope with the increased costs of power retailers due to fluctuations in coal prices and the proliferation of new packages on the customer side that have stagnated or even declined, power retailers have set up energy conservation services on top of the cooling-heating-electricity multi-energy packages to increase their attractiveness to customers while improving their functional security. A schematic diagram of the new business model for power retailers is shown in Figure 1.

**Figure 2:**
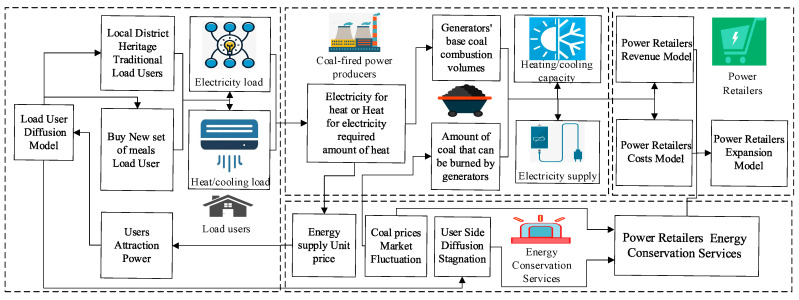
Modeling process of retail strategy for power retailers considering energy conservation services.

A model of the retail strategy of power retailers considering energy guarantee services includes a diffusion process for the power retailers to promote cooling-heating-electricity multi-energy packages to load customers and a comparative analysis process for power retailers with or without energy conservation services. The modeling principle is shown in Figure 2.

The new cooling-heating-electricity multi-energy package, which takes into account energy conservation services, is promoted by power retailers with a generator background among the region’s load customers. Based on the number of traditional customers and customers purchasing new packages, as well as different monitoring data on heat and electricity demand, the power producer uses heat for electricity or electricity for heat to confirm the power producer’s base coal consumption and the unit price of energy supply for different time periods. By comparing the amount of coal that can be burned by the generators, the actual amount of coal burned by the generators and the amount of heat/cooling and electricity supplied by power retailers to the load consumers per hour are known. Based on the amount of energy supplied and the unit price of energy supplied, the cost–benefit model of the power retailers is constructed to form the basis for the expansion of the power retailers. For load customers, the proliferation of new business models on the load side is mainly determined by the attractiveness of the customer influenced by the high or low unit price of thermal electric energy. However, the analysis of the IOT data collection shows that there are cost increases for power retailers with a generator background that occur when the coal price market fluctuates, as well as possible stagnation of diffusion on the user side. In response, power retailers set up energy guarantee services to reduce costs while waiving heating costs and slightly increasing the unit price of electricity supply to meet customer demand, in order to reduce the negative impact of these exceptional circumstances. This will increase the diffusion of the new business model on the customer side, while enhancing the ability of the power retailers to expand and withstand risks.

### 2.2. A System Dynamics Model for Power Retailers with a Generator Background

**Figure 3:**
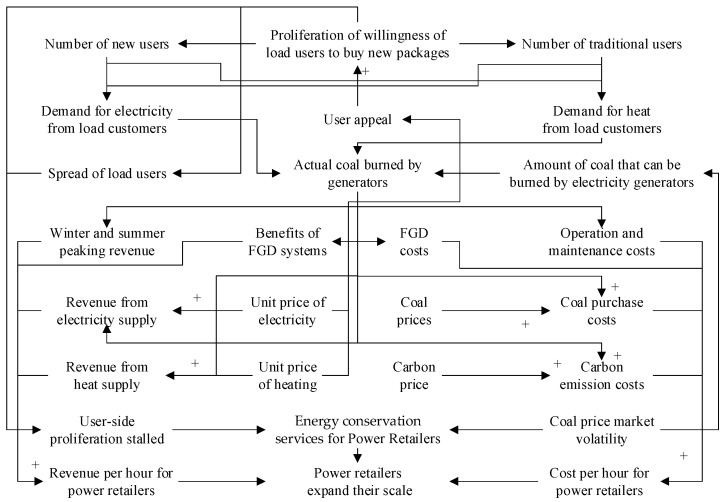
Causal loop diagram.

A system dynamics model [17,18], which can effectively portray the logical relationships of variables and sort out development trends, is used to study a cold-heat-electricity multi-energy commercial package designed by the power retailers considering energy conservation services, and the causal loop diagram is shown in Figure 3. Where “→” in the causal loop diagram indicates the main loop, reflecting the relationship between the long-term extrapolation, the medium-term extrapolation and the short-term extrapolation of the retail behavior of the power retailers and the load consumers to influence each other, “+” indicates a positive correlation between the effects of the variables, and “−” indicates a negative correlation between the variables. In the stock-flow diagram, the rectangular variables are stocks, i.e., cumulative quantities, which characterize the state of the system and provide the basis for decisions and actions; the remaining variables are flows, i.e., rate quantities, which characterizes the rate of stock change. The two variables are logical to each other, forming a system dynamics model that constructs a feedback loop relationship for the retail strategy of power retailers based on a generator background to promote a cold-heat-electricity multi-energy commercial package that considers energy conservation services.

A causal loop diagram of the system dynamics of the power retailers with a generator background is composed of the diffusion behavior of the cold-heat-electricity multi-energy package among load customers and the expansion strategy of the power retailers considering energy conservation services. The power retailers influence the level of customer attractiveness by setting the price of energy conservation, the load users then decide whether to purchase the new type of package or not, thus influencing the number of new type users versus traditional users, i.e., the diffusion of the new type of package on the load side. The generators determine the actual amount of coal to be burned based on energy demand data that monitors the impact of the number of users of both loads, providing a basis for measuring the revenue per hour for power retailers such as electricity supply, heat supply, winter and summer peaking and flotation gypsum, and the cost per hour for power retailers such as coal purchase, operation and maintenance, desulphurization and carbon emissions. Power retailers consider activating energy assurance services to control cost benefits when coal price markets fluctuate versus customer-side proliferation stagnation, which in turn affects the scale of expansion of power retailers.

#### 2.2.1. Power Retailers Cooling-Heat-Electricity Multi-Power Package Model

**Figure 4:**
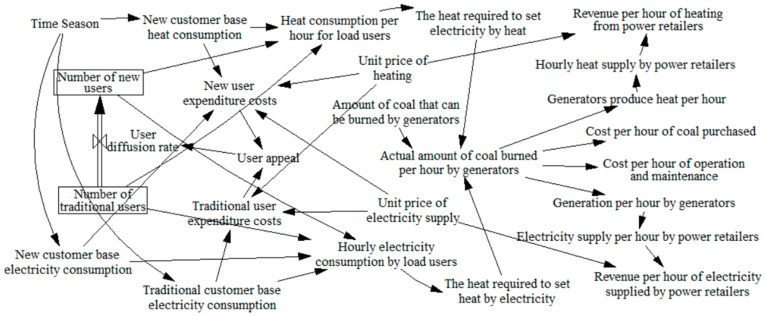
Diffusion stock flow diagram for the cold-heat-electricity multi-energy package.

Power retailers with a power producer background promote cooling-heat-electricity multi-energy packages among load customers in their region. Based on monitoring the heat, cooling and electricity demand of load users in different seasons, and combining the number of new and traditional users, the maximum amount of heat required to meet the heat-to-electricity or electricity-to-heat requirements can be obtained. Taking into account the amount of coal that can be burned by the power producer at this time, the actual amount of coal burned by generators at each hour is determined to meet the energy needs of the load consumers. The power retailers receive hourly revenue from the hourly heat and power supply, net of losses, and pays for the purchase and maintenance costs based on the actual amount of coal burned. Load customers are attracted to the new packages based on a comparison of the cost of the traditional electricity package and the multi-energy package. This in turn influences the diffusion of the cooling-heating-electricity multi-energy package on the customer side. The specific stock flow diagram is shown in Figure 4. The corresponding data are collected through IOT technology and the mathematical model is constructed as shown in Table 1.

#### 2.2.2. Power Retailers Desulphurization and Carbon Emission Model

**Figure 5:**
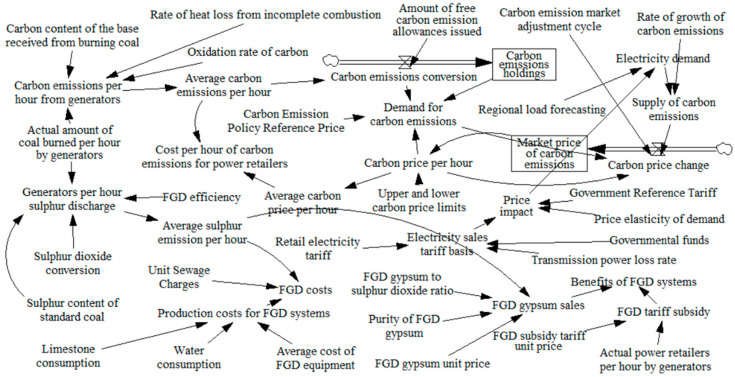
Stock flow diagram of costs and benefits of desulphurization and carbon emissions.

The power retailers burn coal to produce heat and electricity according to the energy needs of the load consumers, and the testing of the sulfur dioxide emission detection of coal-fired units is a necessary link, which is supervised by environmental protection agencies. At the same time, the current carbon market is gradually developing, and power retailers with a generator background should actively participate. Therefore, sulfur emissions and carbon emissions are selected as important contents affecting the retail strategy of power retailers. Based on the carbon and sulfur content of the coal, the power producer measures the hourly carbon and sulfur emissions, respectively, and averages them over a cumulative period of 24 h. Based on carbon market trading, the average hourly carbon price is used to obtain the hourly cost of carbon emissions for the power retailers, taking into account factors such as carbon emissions holdings, carbon emissions demand and carbon prices. Coal-fired units are required to install flue gas desulphurization (FGD) systems in order to emit compliant emissions. Power producers pay for the emissions after FGD on the one hand, and operate the FGD system on the other, selling the by-product FGD gypsum for revenue on top of the production cost of the FGD system, while enjoying the FGD tariff subsidy. The specific stock flow diagram is shown in Figure 5. The corresponding data are collected through IOT technology and the mathematical model is constructed as shown in Table 2.

#### 2.2.3. Power Retailers Energy Conservation Service Model

**Figure 6:**
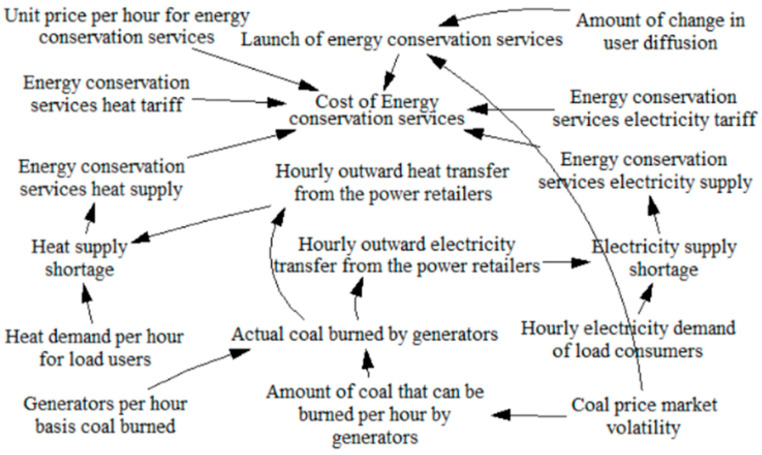
Stock flow map of energy conservation services.

The power retailers monitor its costs, revenues, scale of expansion and the proliferation of new packages through IOT data collection, interconnection and analysis technologies. In response to increased costs in the event of market fluctuations in coal prices and stagnation in the proliferation of new packages on the customer side, the energy guarantee service is also activated. The shortfall will be made up by the heat and power supply capacity of the energy conservation service to meet the demand of the load users based on the heat and power tariff of the energy conservation service, by measuring the hourly external heat and power supply of the power retailers and the hourly heat and power demand of the load users, respectively. The specific stock flow diagram is shown in Figure 6. The corresponding data are collected through IOT technology and the mathematical model is constructed as shown in Table 3.

## 3. Retailing Strategies for Power Retailers with a Generator Background

A system dynamics model for the power retailers with a generator background is constructed based on the collection and monitoring of data on the costs, revenues and expansion of the power retailers and the proliferation of load customers in the market environment. Based on the monitoring data of the electricity and heat demand of the load users in the region, the power retailers will purchase the corresponding amount of coal for heating and supplying electricity through the determination process of “electricity for heat” or “heat for electricity”, taking into account the amount of coal that can be burned by the power producer at this time, paying the hourly cost of coal purchase and unit operation and maintenance costs and obtaining revenue for heat and power supply. For excess heat and electricity to be stored through hot water storage tanks and storage power stations, power retailers participate in the peaking market in winter and summer to obtain peaking revenue, and generators recycle the stored waste heat to improve resource utilization efficiency. As a result of the goal of green and sustainable development, power producer has installed FGD systems and participated in the carbon emissions market. In the process of FGD of coal-fired flue gas, FGD systems need to invest in the production costs of the system, while the by-products produced, i.e., FGD gypsum, are sold for revenue, on the other hand, power producers actively respond to the “double carbon” target by participating in carbon trading and paying carbon emission costs. In response to coal price market fluctuations and diffusion standstill situations, power retailers set up energy conservation services and bear the costs of energy conservation services. The cost–benefit of power retailers affects their expansion. The power retailers promote the cold-heat-electricity multi-energy package to load customers, whose heat tariff is the main influence on customer-side proliferation.

### 3.1. Cost Model for Power Retailers

The cost per hour of coal purchased by the power producer, the cost of operation and maintenance of the unit and storage equipment, the cost of carbon emissions from the production and discharge of the FGD system and the cost of energy assurance services are mainly considered. Of these, the operation and maintenance costs of energy storage equipment include the hourly operation and maintenance costs of hot water storage tanks and the hourly charging and discharging operation and maintenance costs of energy storage plants, as follows:

(1) $$C_{g}^{h}=C_{M}^{h}+O_{G}^{h}+O_{R}^{h}+O_{E}^{h}+C_{T}^{h}+C_{S}^{h}+C_{C}^{h}+C_{B}^{h}+C_{g0}^{h}$$

where, $C_{g}^{h}$ is the cost per hour of the power retailers. $C_{M}^{h}$ is the cost per hour of coal purchased by the coal-fired power producer. $O_{G}^{h}$, $O_{R}^{h}$ and $Q_{E}^{h}$ are the cost per hour of operation and maintenance of generating units, hot water storage tanks and energy storage plants, respectively. $C_{S}^{h}$ and $C_{C}^{h}$ are the cost per hour of sulfur dioxide emissions and carbon emissions, respectively. $C_{T}^{h}$ is the cost per hour of production of the FGD system. $C_{B}^{h}$ is the cost per hour of the energy assurance service of the power retailers. $C_{\mathrm{g0}}^{h}$ is the initial value of the cost per hour of the power retailers.

#### 3.1.1. Cost per Hour of Coal Purchased by Coal-Fired Generators

Based on real-time monitoring data of the heat demand and electricity demand of load users at each hour, coal-fired power producers will convert the greater of heat for electricity or electricity for heat for coal-fired heat to obtain the amount of coal purchased at each hour, as follows:

(2) $$Q_{h}^{h}=\frac{Q_{r}^{h}}{h_{r}}$$

(3) $$Q_{e}^{h}=\frac{L_{r}^{h}\cdot q_{e}}{h_{e}}$$

where, $Q_{e}^{h}$, $Q_{h}^{h}$ is the heat requirement for electricity for heat and heat for electricity, respectively. $Q_{r}^{h}$ is the actual heat demand per hour of the load user. $L_{r}^{h}$ is the actual hourly demand for electricity by the user. $h_{r}$, $h_{e}$ is the heat and electricity consumption, respectively. $q_{e}$ is the heat required per unit of electricity generated.

(4) $$M_{b}^{h}=\frac{\max\left(Q_{h}^{h},Q_{e}^{h}\right)}{q_{M}}$$

(5) $$M_{r}^{h}=\min\left(M_{k}^{h},M_{b}^{h}\left(1+\alpha_{M}\right)\right)$$

where, $q_{M}$ is the amount of heat generated per unit of coal burned. $M_{b}^{h}$ is the base hourly coal use of the electricity producer. $M_{r}^{h}$ is the actual amount of coal used by the generators per hour. $M_{k}^{h}$ is the amount of coal available for combustion by the generators per hour. $\alpha_{M}$ is the coal reserve ratio of the generators.

(6) $$C_{M}^{h}=M_{r}^{h}\cdot P_{M}$$

where, $P_{M}$ is the actual unit price of standard coal.

#### 3.1.2. Operating and Maintenance Costs per Hour for Units and Energy Storage Equipment

Based on the generation and storage capacities of coal-fired generating units, energy storage plants and hot water storage tanks, the unit and storage equipment operation and maintenance costs per hour can be obtained based on the respective operation and maintenance cost unit prices, as follows:

(7) $$O_{G}^{h}=C_{M}^{h}\cdot o_{g}$$

(8) $$O_{E}^{h}=D_{f}^{h}\cdot o_{e}$$

(9) $$O_{R}^{h}=Q_{f}^{h}\cdot o_{r}$$

where, $o_{g}$, $o_{e}$, $o_{r}$ is the share of operation and maintenance costs for power producers and the unit operation and maintenance costs for energy storage plants and hot water storage tanks, respectively. $D_{f}^{h}$, $Q_{f}^{h}$ is the amount of discharge per hour and the amount of heat discharged from the storage plant and hot water storage tank, respectively.

(10) $$Q_{f}^{h}=\min\{E_{Q}^{h},Q_{\max}^{h}\}$$

(11) $$E_{Q}^{h}=\sum_{t=0}^{T}(Q_{i}^{h}-Q_{f}^{h})$$

(12) $$Q_{i}^{h}=\min\{U_{h}^{h},Q_{\max}^{h}\}$$

(13) $$U_{h}^{h}=\max\left(B_{h}^{h},0\right)$$

where, $E_{Q}^{h}$ is the heat stored per hour in the hot water storage tank. $Q_{\max}^{h}$ is the maximum amount of heat stored in the hot water storage tank. $Q_{i}^{h}$ is the amount of heat charged to the hot water storage tank. $U_{h}^{h}$ is the surplus of heat produced by the electricity producer. $B_{h}^{h}$ is the heat supply deviation per hour of the power retailers.

#### 3.1.3. Cost per Hour of Production and Discharge Carbon Emissions from Desulphurization Systems

There are sulfur dioxide pollution and carbon dioxide emissions from the burning of coal by the power producers who install desulphurization systems to reduce sulfur dioxide emissions and pay carbon emission fees to the carbon market, as follows:

(14) $$C_{S}^{h}=\frac{F_{s}^{d}}{24}\cdot P_{s}$$

where, $P_{S}$ is the sulfur dioxide emission charge. $F_{s}^{h}$ is the sulfur emission per hour.

(15) $$F_{s}^{d}=\sum_{t=0}^{\mathrm{24}}F_{s}^{h}$$

(16) $$F_{s}^{h}=2\cdot M_{r}^{h}\cdot M_{S}\cdot \lambda_{S}\cdot (1-\beta_{T})$$

where, $M_{S}$ is the proportion of sulfur in the standard coal. $\lambda_{S}$ is the sulfur dioxide conversion rate. $\beta_{T}$ is the desulphurization efficiency; $F_{s}^{d}$ is the daily sulfur emission.

(17) $$C_{T}^{h}=C_{T0}^{h}+T_{W}^{h}+T_{C}^{h}$$

where, $C_{T0}^{h}$ is the average cost of FGD equipment. $T_{W}^{h}$, $T_{C}^{h}$ is the cost of water consumption and limestone consumption per hour of the FGD system, respectively.

(18) $$T_{W}^{h}=0.15\cdot D_{e}^{h}\cdot P_{W}$$

(19) $$T_{C}^{h}=0.01\cdot D_{e}^{h}\cdot P_{C}$$

(20) $$D_{e}^{h}=\frac{M_{r}^{h}}{m_{e}}$$

where, $D_{e}^{h}$ is the actual amount of electricity generated by the generators per hour. $m_{e}$ is the amount of coal required per unit of electricity generated. $P_{W}$, $P_{C}$ is the unit price of water and limestone, respectively.

(21) $$C_{C}^{h}=P_{C}^{h}\cdot F_{C}^{h}$$

(22) $$F_{C}^{h}=\frac{1}{\mathrm{24}}\sum_{t=0}^{\mathrm{24}}(3.668\cdot M_{r}^{h}\cdot (1-\eta_{r})\cdot \Phi_{C}\cdot \varepsilon_{C})$$

where, $F_{C}^{h}$ is the average hourly carbon emission. $\eta_{r}$, $\Phi_{C}$, $\varepsilon_{C}$ is the heat loss rate from incomplete combustion, the carbon content of the received base from burning coal and the oxidation rate of carbon, respectively. $P_{C}^{h}$ is the average hourly carbon price.

#### 3.1.4. Cost per Hour for Energy Assurance Services

The cost per hour of energy conservation services is the product of the shortfall in cold-heat-electricity and the price of energy conservation in response to the increased costs that occur in the event of market fluctuations in coal prices and the stagnation of the proliferation of new packages on the customer side. This is indicated by changes in the number of new customers, as follows:

(23) $$C_{B}^{h}=X_{h}^{h}\cdot I_{h}+X_{e}^{h}\cdot I_{e}$$

where, $C_{B}^{h}$ is the cost per hour of energy conservation services. $I_{h}$, $I_{e}$ is the unit prices for heat and electricity supply for energy conservation services, respectively. $X_{h}^{h}$, $X_{e}^{h}$ is the unit prices of heat and electricity for energy conservation services per hour, respectively.

(24) $$X_{h}^{h}=\left|\min\left(B_{h}^{h},0\right)\right|$$

(25) $$X_{e}^{h}=\left|\min\left(B_{e}^{h},0\right)\right|$$

where, $B_{e}^{h}$ is the amount of deviation per hour of electricity supplied by the power retailers.

(26) $$B_{h}^{h}=(H_{M}^{h}-H_{e}^{h})\cdot (1-\mu_{h})\cdot (1-\Delta_{h})-Q_{r}^{h}$$

(27) $$B_{e}^{h}=S_{e}^{h}\cdot (1-\mu_{e})\cdot (1-\Delta_{e})-L_{r}^{h}$$

where, $\mu_{h}$, $\mu_{e}$ is the ratio of heat to electricity used by the power producer’s plant. $\Delta_{h}$, $\Delta_{e}$ is the rate of heat and electricity loss in transmission, respectively. $H_{M}^{h}$ is the amount of heat produced by the power producer per hour of coal combustion. $H_{e}^{h}$ is the heat required by the power producer to generate electricity. $S_{e}^{h}$ is the actual amount of electricity generated by the power producer per hour.

(28) $$H_{M}^{h}=M_{r}^{h}\cdot q_{M}$$

(29) $$H_{e}^{h}=S_{e}^{h}\cdot q_{e}-E_{Q}^{h}$$

(30) $$S_{e}^{h}=\frac{M_{r}^{h}}{m_{e}}$$

### 3.2. Revenue Model for Power Retailers

Revenue per hour from participation in peaking, revenue per hour from sale of electricity and heat and revenue per hour from sale of desulphurization gypsum in winter and summer are mainly considered. Of these, the revenue per hour of participation in peaking is related to the charging and discharging of the energy storage plant, as follows:

(31) $$Y_{g}^{h}=Y_{E}^{h}+Y_{H}^{h}+Y_{S}^{h}+Y_{T}^{h}+Y_{g0}^{h}$$

where, $Y_{g}^{h}$ is the hourly revenue of the power retailers. $Y_{T}^{h}$ is the revenue per hour of participation in peaking during winter and summer. $Y_{E}^{h}$ and $Y_{H}^{h}$ is the revenue per hour from the sale of electricity and heat by the power retailers, respectively. $Y_{S}^{h}$ is the revenue per hour of FGD system operation and production. $Y_{g0}^{h}$ is the initial value of revenue per hour for the power retailers.

#### 3.2.1. Revenue per Hour from Participation in Peaking in Winter and Summer

The power retailers make the power producer burn coal to produce heat and electricity according to the load users’ energy demand, and part of the load users’ electricity demand is provided by cooling and heating in winter and summer. After satisfying the actual electricity consumption of the load users in the region, the remaining electricity is stored in the energy storage power station, which participates in peaking in different time periods according to the different peaking requirements in the peaking market, as follows:

(32) $$Y_{T}^{h}=P_{T}\cdot E_{D}^{h}$$

where, $P_{T}$ is the peak price per unit of electricity. $E_{D}^{h}$ is the hourly storage capacity of the energy storage plant.

(33) $$D_{f}^{h}=\min\{E_{Q}^{h},E_{\max}^{h}\}$$

(34) $$E_{D}^{h}=\sum_{t=0}^{T}(D_{i}^{h}-D_{f}^{h})$$

(35) $$D_{i}^{h}=\min\{U_{e}^{h},E_{\max}^{h}\}$$

(36) $$U_{e}^{h}=\max\left(B_{e}^{h},0\right)$$

where $E_{\max}^{h}$ is the maximum storage capacity of the energy storage plant. $D_{i}^{h}$ is the charging capacity of the energy storage plant. $U_{e}^{h}$ is the surplus of electricity generated by the power producer.

#### 3.2.2. Revenue per Hour from Sale of Electricity and Heat

The electricity supply revenue of the power retailers is the product of the actual electricity consumption of the load customers in the region and the retail electricity price. The heat supply revenue is the product of the actual heat consumption of the load customers in the region and the unit price of heat, as follows:

(37) $$Y_{E}^{h}=L_{r}^{h}\cdot P_{E}$$

(38) $$Y_{H}^{h}=Q_{r}^{h}\cdot P_{H}$$

where, $P_{E}$, $P_{H}$ is the retail tariff and the unit price of heating, respectively.

#### 3.2.3. Revenue per Hour of FGD System Operation and Production

The power retailer receives revenue from by-product sales and tariff subsidies through the FGD equipment, as follows:

(39) $$Y_{S}^{h}=A_{E}^{h}+A_{S}^{h}$$

(40) $$A_{E}^{h}=S_{e}^{h}\cdot A_{s}$$

(41) $$A_{S}^{h}=\frac{F_{s}^{h}\cdot \omega_{s}}{\delta_{G}}\cdot P_{G}$$

### 3.3. Expansion Model for Power Retailers

The costs and benefits of the power retailers are considered primarily, while the attractiveness of the load customers is used as a reference to form a model for the expansion of the power retailers.

#### 3.3.1. User Appeal

Customer attractiveness is the level of acceptance of the new commercial package by load customers participating in the cooling-heating-electricity multi-energy model. The power retailers determine the attractiveness of the customer based on the ratio of the load customer’s traditional heat and cooling costs to its new package purchase heat and cooling costs, as follows:

(42) $$M_{y}=\frac{J_{T}-J_{N}}{J_{T}}$$

where, $M_{y}$ is the attractiveness of the new package to the customer. $J_{T}$, $J_{N}$ is the cost of conventional cooling heat production for load customers versus the cost of purchased heat and cooling energy for the new package, respectively.

#### 3.3.2. Expansion Rate of Power Retailers

The power retailers use the profit of the difference between costs and revenues as an important basis for the power retailers’ expansion, with reference to customer attractiveness, but also limited by the size of the market in the region, as follows:

(43) $$K_{g}=\left(1+\frac{Y_{g}^{h}-C_{g}^{h}}{C_{g}^{h}}\right)\cdot \left(1+M_{y}\right)\cdot \left(1-\frac{n_{g}}{\theta }\right)$$

where, $K_{g}$ is the expansion rate of power retailers. $n_{g}$ is the size of power retailers. θ is the size of the market.

#### 3.3.3. Size of Power Retailers

The size of the power retailers is influenced by the expansion rate of the power retailers and the initial size. The INTEG function is used to integrate the expansion rate of the power retailers, using the initial size as the initial value, to find the cumulative quantity of the size of the power retailers, as follows:

(44) $$n_{g}=INTEG\left(K_{g},n_{g0}\right)$$

where, $n_{g0}$ is the initial size of the power retailers.

### 3.4. Load User Diffusion Model

The number of benefits that a load customer can obtain from choosing a cooling-heating-electricity multi-energy package compared to a traditional electricity package is mainly taken into account, resulting in a load customer diffusion model.

#### 3.4.1. New and Traditional Package User Expenses

The hourly cost of the new package is calculated for load users who choose the new package by using the hourly electricity and heat consumption and the electricity and heat tariffs set by the power retailers, respectively, as follows:

(45) $$Z_{N}^{h}=R_{H}^{h}+R_{E}^{h}$$

where, $Z_{N}^{h}$ is the cost per hour for new package users. $R_{H}^{h}$ and $R_{E}^{h}$ is the cost per hour for new package users for heat and electricity, respectively.

(46) $$R_{E}^{h}=P_{E}\cdot (1+b_{E})\cdot E_{N}^{h}$$

(47) $$R_{E}^{h}=P_{H}\cdot (1+b_{H})\cdot H_{N}^{h}$$

where, $b_{E}$ and $b_{H}$ are fluctuations in heat and electricity consumption, respectively. $E_{N}^{h}$ and $H_{N}^{h}$ are the hourly base electricity and heat consumption of the new package users, respectively.

Load customers who choose the traditional package calculate the hourly traditional package customer expenditure cost by using the hourly electricity consumption and the tariff set by the power retailers, as follows:

(48) $$Z_{T}^{h}=P_{E}\cdot (1+b_{E})\cdot E_{T}^{h}$$

where, $Z_{T}^{h}$ is the cost per hour for traditional package customers. $E_{T}^{h}$ is the base hourly electricity consumption for traditional package customers.

#### 3.4.2. Expansion Rate of Power Retailers

The power retailers promote cold-heat-electricity multi-energy packages on the customer side. The strength of the discount paid for the new package and the traditional package in meeting the customer’s energy needs is an important basis for customer-side proliferation, as follows:

(49) $$K_{y}=\left(1+\frac{Z_{T}^{h}-Z_{N}^{h}}{Z_{T}^{h}}\right)\cdot \left(1+M_{y}\right)+\left(1+\zeta_{y}\right)$$

where, $K_{y}$ is the load-user diffusion rate. $\zeta_{y}$ is the load-user willingness factor.

#### 3.4.3. Number of Load User Diffusion

The number of load users selected to participate in the cooling-heat-electricity multi-energy package diffusion is influenced by the diffusion rate, again calculated using the INTEG function for integration, as follows:

(50) $$n_{y}=INTEG\left(K_{y},n_{y0}\right)$$

where, $n_{y0}$ is the initial number of load users in the region.

## 4. Case Analysis

### 4.1. Case Basic Data

#### 4.1.1. Parameters Related to Power Retailers with a Generator Background

**Figure 7:**
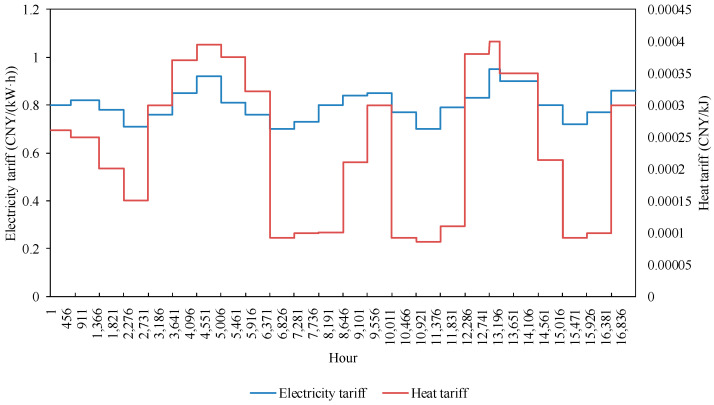
Change in electricity price and heat price.

Power retailers with a generator background fully utilize their own CHP advantages to promote cooling-heat-electricity multi-energy packages on the customer side, with retail electricity and heating unit prices, as shown in Figure 7, coal-fired unit-related parameters [21,22], as shown in Table 4, FGD system-related parameters, as shown in Table 5, and carbon emission market-related parameters [23], as shown in Table 6.

#### 4.1.2. Load User Related Parameters

The load users in the region participate in the cooling-heating-electricity multi-energy package, taking their relevant parameters [24], as shown in Table 7.

**Figure 8:**
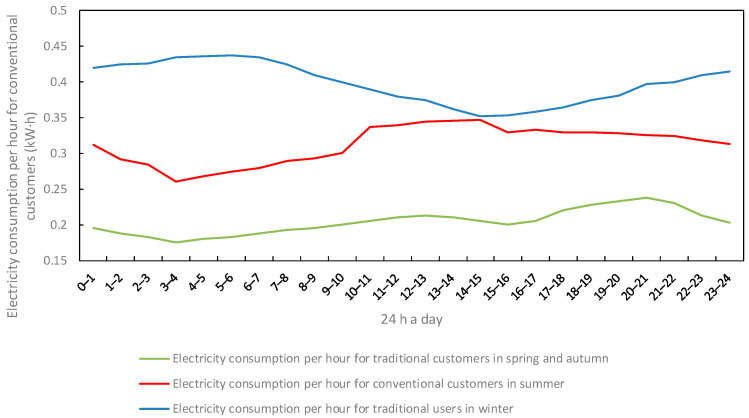
Change in electricity consumption of traditional load users.

**Figure 9:**
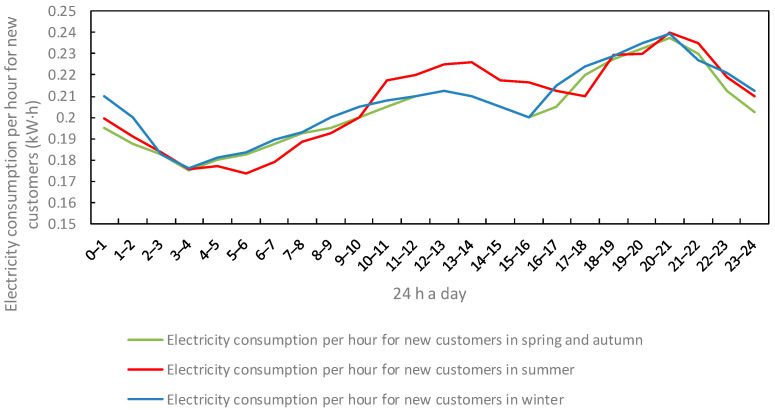
Change in electricity consumption of new load users.

**Figure 10:**
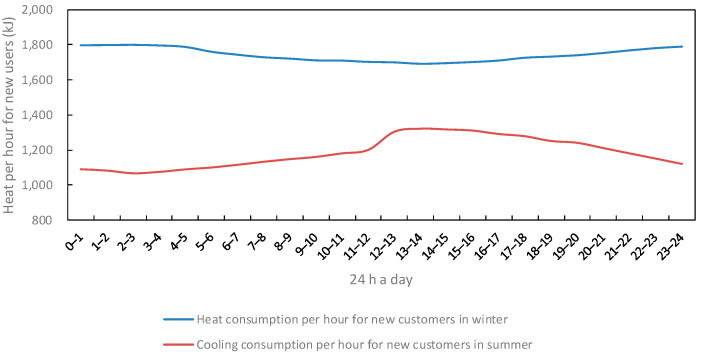
Change in heat consumption of new load users.

The variation in customer electricity and heat consumption over time and seasons for 24 h a day for traditional or participating load users in the region is shown in Figure 8, Figure 9 and Figure 10.

### 4.2. Analysis of Demand for Energy Assurance Services for Power Retailers

Using Vensim software, this paper describes an example of power retailers with a generator background promoting a cooling-heating-electricity multi-energy package to load customers in the wholesale and retail electricity markets. This is to attract load customers to participate in the multi-energy cooling and heating package and to increase the size of the power retailers in the market, while generating revenue for the load customers. For this, the total simulated hours are 17,280, or 720 days, or 24 months, if calculated on 30 days per month.

#### 4.2.1. Analysis of Load User Diffusion Model Runs

**Figure 11:**
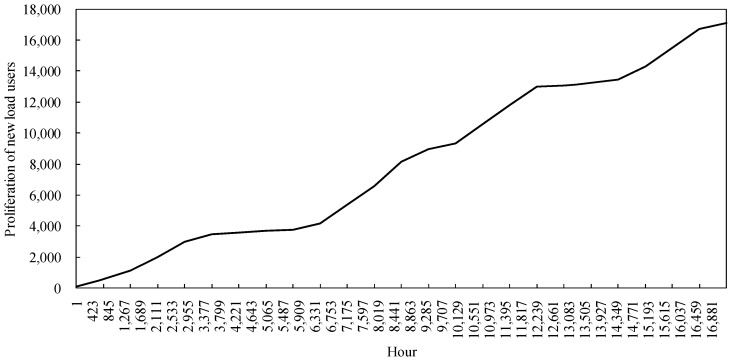
Diffusion of load users.

The statistical variation of the 17,280 h, simulated for the load customer diffusion model in the example, is shown in Figure 11. Among them, the number of load customers choosing to participate in the cooling-heating-electricity multi-energy package shows an overall increasing trend, which can indicate that the power retailers providing cooling, heating and electricity multi-energy services for traditional customers is welcomed by customers. However, at the same time, the heat price is high during the peak cooling hours in summer due to the constraints of the residual cooling power of the cooling stations and the need for the power retailers to ensure their own revenue, making it possible for there to be a stagnation of customer-side diffusion during the hours 3687 to 5775 and 12,238 to 14,209, namely in the summer months of June to August each year. In winter, the user-side diffusion rate is still lower than in spring and autumn due to the coal price constraint. In addition, the graph shows that during the simulation of the example, the spread of load users shows a zigzag upward trend, but at the end the number of spreads choosing the new business model is less than half of the total number of users in the region. The reason for this is that power retailers with a generator background set higher heat prices to maintain their own revenue in a market where coal prices fluctuate and rise.

#### 4.2.2. Analysis of Power Retailers Cost Model Runs

**Figure 12:**
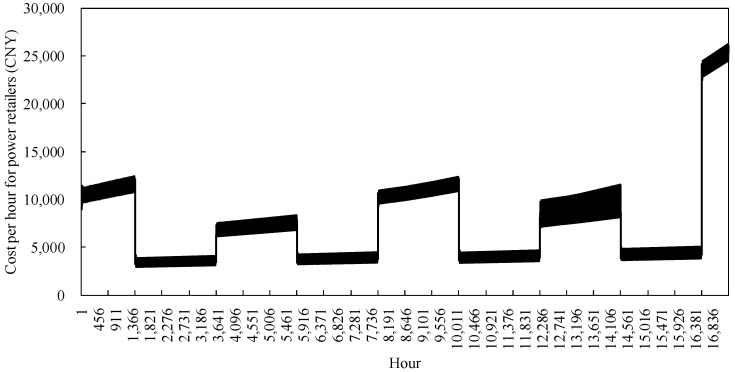
Cost of power retailers.

The cost model simulation of the power retailers in the example monitors changes, as shown in Figure 12. In the spring and autumn, when customers use less heat and cold, the power retailers with a generator background are mainly responsible for the electricity demand of load customers, which is less costly, but at this time, the coal-fired heat is only used for power generation, with some waste heat being recycled. During the peak energy consumption hours in the summer, the demand for electricity from traditional users is high, and the demand for electricity from load users who choose the cold-heat-electricity multi-purpose package does not rise significantly. However, higher heat prices have led to a stagnation in the proliferation of new load users, resulting in a slow trend in the cost of electricity sold during the summer months. Still, costs are higher throughout the summer season than in spring and autumn. During the peak heating hours in winter, the cost of heat production for traditional users using air conditioning is higher than the cost of heat purchase for the new package, which is more attractive to users and generators, even though they have made full use of the CHP mechanism to improve the efficiency of resource utilization. However, the cost of coal purchase is still increased to meet the energy demand of load customers, thus causing a rapid increase in cost per hour for the power retailers.

Throughout the simulation of the calculations, in the first year, the cost of the power retailers is low in spring and autumn, with hourly cost fluctuations of no more than ¥15,000 in summer and winter. In the second year, nearly three in ten traditional customers chose to participate in the cooling-heat-electricity multi-energy package, resulting in an increase in the amount of heat and cooling supplied. While taking full advantage of cogeneration production, the limitations of pipeline transport and unit cycling have led to an upward trend in coal purchases by power producers, especially in the second winter when the costs of the power retailers increase significantly during peak energy consumption hours.

#### 4.2.3. Analysis of Power Retailers Revenue Model Runs

**Figure 13:**
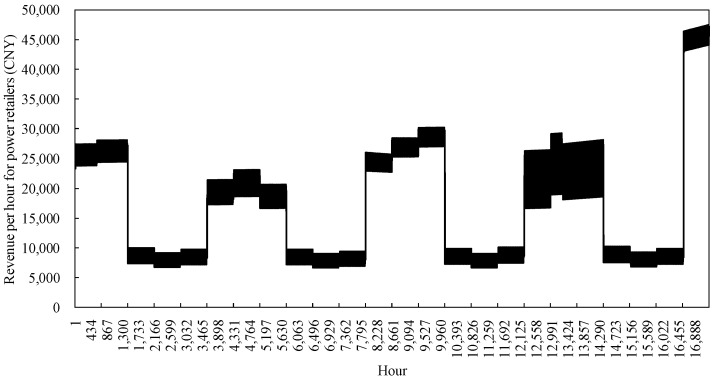
Earnings of power retailers.

The revenue model for the power retailers in the example monitors changes in revenue in a similar way to changes in costs, as shown in Figure 13. Revenues are mainly affected by the retail tariff in spring and autumn, when customer demand for heat is much lower than for electricity, while the low valley tariff during periods of abundant supply results in lower hourly revenues for the power retailers in spring and autumn. In the summer and winter months when customer demand for energy is high, generators use cogeneration to offer heat and power packages to customers, which, on the one hand, improves the efficiency of resource utilization. On the other hand, preferential combination packages attract customers to participate and higher peak hourly heat prices generate more revenue for the power retailers. However, stagnation in the spread of customers results in significantly lower revenues in the summer than in the winter, while the overall trend of growth in load customers allows the revenue of the power retailers to increase in each season of the second year compared to the first year. The revenue curve of the power retailers fluctuate more at the hourly level compared to the cost curve due to changes in the attractiveness of the heat price to customers at various times of the year. In particular, during the summer and winter peak periods, higher heat prices will directly reduce customer demand for energy, making the new package less attractive to customers and thus affecting the hourly revenue of the power retailers.

#### 4.2.4. Analysis of Power Retailers Expansion Model Runs

**Figure 14:**
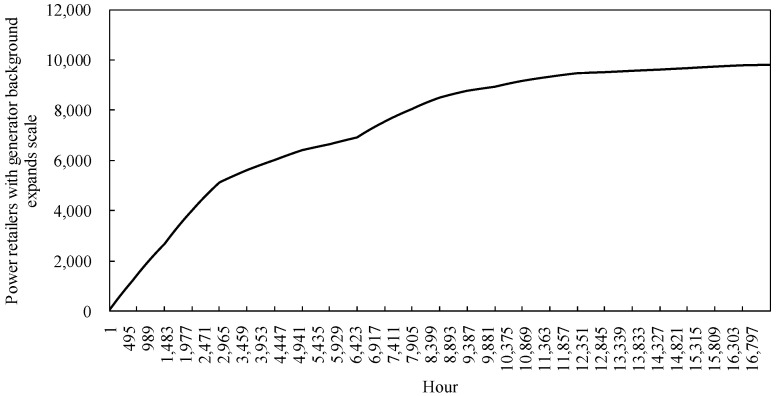
The expansion scale of power retailers.

The expansion model of the power retailers in the example simulates the change in size, as shown in Figure 14. Power retailers with a generator background take advantage of cogeneration based on the energy needs of load users. Electricity and heat tariffs that maintain their own revenue are set to attract more customers to participate in the investment. The size of the power retailers showed an initial upward trend, expanding rapidly as more load users participated in the investment. However, there are constraints such as market saturation and the proportion of customers participating in the cold-heat-electricity multiple-energy model approaching its upper limit. The power retailers show a trend of slowing down their expansion and eventually levelling off. In addition, the graph shows that the expansion curve of the power retailers decreases in speed and slopes down during the simulated hours 3601 to 6682, when demand for cooling from load customers is high. The reason for this is that the higher heat prices at this time cause the proliferation of customers to stagnate, making it impossible for the power retailers to gain more revenue by increasing its coal purchase costs to meet customer demand, which in turn affects its expansion.

### 4.3. Operation of Energy Conservation Services for Power Retailers Considering Market Fluctuations in Coal Prices

**Figure 15:**
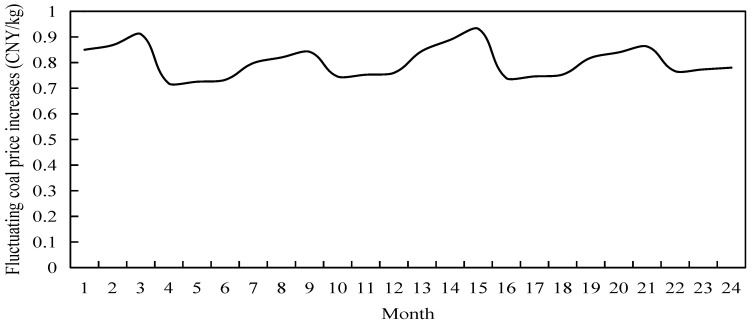
Monthly fluctuations of coal price rise.

The cold-heat-electricity multi-energy packages promoted by generators rely on thermal coal-fired units for cogeneration, thus ensuring multi-energy supply to load users. However, at this stage, the overseas supply of power coal is decreasing, and although the main coal-fired production areas are implementing supply protection policies, the tight supply and demand pattern remains unchanged, and coal prices are still running high within the price limit level. Therefore, this example simulates the fluctuating increase in coal prices by counting the change in the average social coal consumption and using the benchmark coal price as the basis, as shown in Figure 15.

Combined with the actual power coal price changes [24], it can be seen that spring and autumn are low price periods, while summer and winter coal prices show an upward phase. Due to the strong demand for heating coal in winter, coal prices rise more than in summer due to supply and demand. Therefore, for power retailers with a power producer background, even if they give full play to cogeneration and waste heat cooling technologies in the summer and winter, higher coal prices inevitably place a greater cost burden on power retailers during peak energy consumption hours for load users. In this regard, power retailers can reduce the cost of coal purchased by power producers by purchasing energy conservation services.

Vensim software is used to simulate the cost per hour of energy supply for the power retailers, considering an energy conservation service in response to fluctuating coal price increases in the market. Instead of purchasing additional coal reserves during the summer and winter months when coal prices are high, the energy conservation service provider will provide the energy needs of the remaining load customers for electricity consumption. Compared to the cost of the power producer’s own coal purchase for coal-fired generation, the energy supply fee paid to the energy conservation service provider effectively mitigates the hourly cost of the power retailers during peak coal price periods, thereby accelerating the expansion of the power retailers.

The parameters related to the energy conservation service of the power retailers with a generator background are shown in Table 8. The power retailers pay a monthly fee to the energy conservation service provider for the daily costs of energy assurance. When market fluctuations in coal prices lead to higher costs for the power retailers and profits are driven by higher energy charges paid by load users, the power retailers consider purchasing electricity and heat from the energy conservation service provider to supply its customers, while cutting its own costs for purchasing coal-fired capacity.

**Figure 16:**
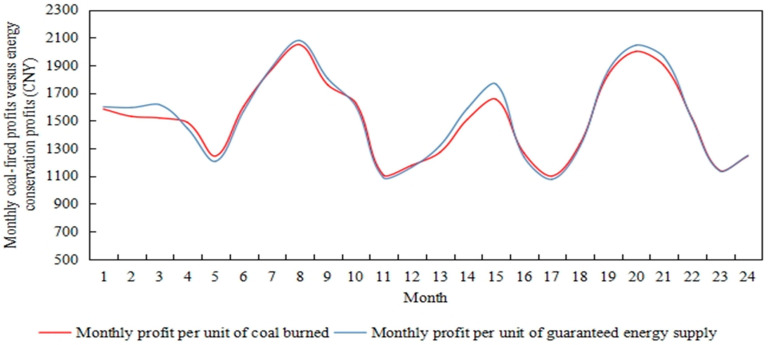
The comparison of monthly unit coal-fired profit and energy supply profit of power retailers.

In this regard, the monthly profit per unit of coal burned by the power retailers is compared with the profit from energy conservation. On the one hand, the difference between the revenue generated by burning one kilogram of coal per hour purchased by the electricity producer and the cost of operating and maintaining the coal is the profit per hour, simulated for 720 h, i.e., one month in total, forming the monthly profit per unit of coal for the power retailers. On the other hand, the power retailers purchase the same amount of thermal electricity from the energy assurance service provider to supply to customers, again simulating a cumulative month to form a monthly profit per unit of energy assurance for the power retailers, as shown in Figure 16 for comparison.

As can be seen from Figure 16, the monthly unit coal profits of power retailers and energy supply profits show seasonal fluctuations, and their trends are broadly similar. Combined with the fluctuating upward changes in coal prices in Figure 15, in spring and autumn when coal prices are in the valley of fluctuation, generators have ample supply capacity and supply load users to obtain revenue to better fill the cost of coal purchase, unit operation and maintenance, and carbon emission desulphurization costs. The energy conservation service provider is not able to control costs for the power retailers at this time, which pays the day-to-day costs and uses the energy assurance service as an emergency back-up. In the summer and winter seasons, as coal prices are on an upward trend, particularly during the winter months when supply and demand are tightly balanced due to factors such as centralized heating, the uplift in coal prices is significant. Compared to the power producers’ own coal purchasing capacity, the profit of the Energy Assurance Service is increased by a minimum of 1% and a maximum of 5% in winter, and by a maximum of 2.8% and a minimum of 0.8% in summer. At this point, the energy guarantee service is an effective way of controlling the costs of the power retailers. After the power retailers have decided on the hourly base coal consumption of the power producer based on the energy demand of the load consumers by adopting a heat-for-electricity or electricity-for-heat approach, the proportion of coal storage reserve and energy loss is cut back and a large proportion of energy supply adequacy is no longer considered. Thus, the amount of increase in coal purchase costs is reduced in the summer and winter months.

**Figure 17:**
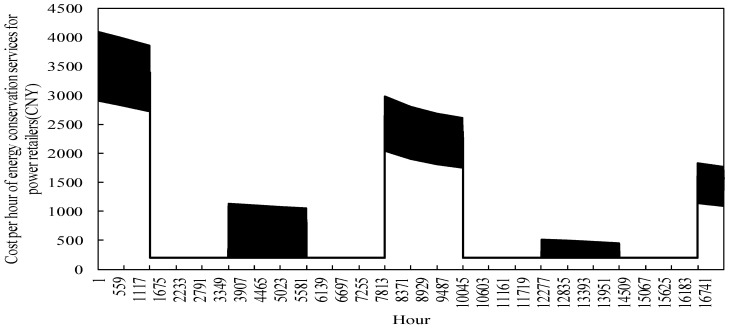
The change of energy conservation service cost per hour in power retailers.

The change in the cost of energy assurance services per hour for power retailers with a generator background, taking into account fluctuations in the coal price market, is shown in Figure 17. As can be seen from the graph, in the simulation, the cost of energy assurance services varies seasonally, and is significantly higher in the first year than in the second. The reason for this is that in the first year, traditional load users still make up the majority of the customers supplied by the power retailers, and power retailers only benefit from the sale of electricity per unit of coal burned, which is not enough to enable the power retailers to grow rapidly in relation to its costs. As a result, the power retailers introduced energy guarantee services to cut costs based on the operation of coal-fired units. In the second year, as the spread of cooling-heat-electricity multi-energy packages becomes wider on the customer side, more load users opt for the new packages. The generators take full advantage of cogeneration technology to expand the revenue from heat sales on top of the existing electricity sales, increasing the revenue per hour for the power retailers and thus reducing the use of energy conservation services. At the same time, the faster increase in coal prices during the winter months makes the scale of expansion from the use of energy assurance services more significant during the winter months. As a result, the amount of energy used by power retailers for energy assurance services is significantly higher in winter than in summer, which also results in higher hourly costs for energy assurance than in summer. At the same time, the energy demand of load customers varies throughout the 24 h of the day, resulting in fluctuations in the hourly cost of energy assurance services for power retailers.

**Figure 18:**
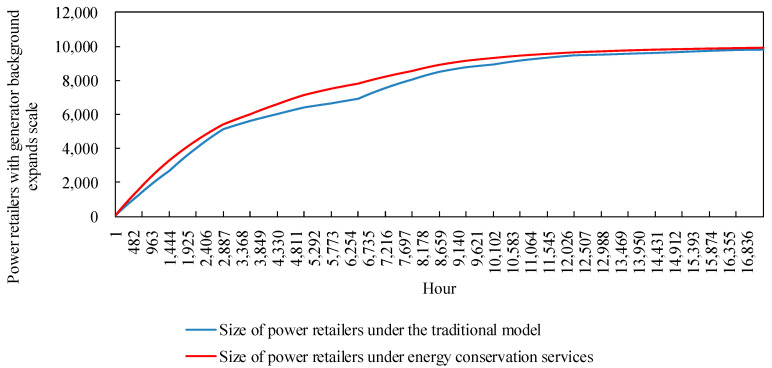
Comparison of the expansion of power retailers considering coal price fluctuations and energy conservation services.

In view of the seasonal peaks and valleys in the coal price market, the power retailers with a power producer background promote the cold-heat-electricity multi-energy package to load customers. On the one hand, the CHP technology was fully utilized, and the efficiency of resource utilization was improved. On the other hand, energy conservation service providers were introduced, and power retailers purchased energy conservation services during periods of rising coal prices. The additional coal purchases by the power retailers was cut and the cost outlay was reduced by purchasing energy conservation services. A comparison of the expansion of power retailers considering market fluctuations in coal prices and energy conservation services is shown in Figure 18.

Based on the comparison of the scale of expansion of the power retailers under the traditional model, with the scale of expansion of the power retailers considering energy guarantee services in Figure 18, it can be clearly observed that by setting up an energy guarantee service, the power retailers are able to reduce the cost of coal purchased by the power retailers by purchasing energy guarantee services during the summer and winter months when coal prices rise. This allows them to effectively control the expansion rate of the power retailers when coal prices fluctuate in the market. Combined with the stagnation of load users in the summer months in Figure 11, the energy guarantee service still allows the power retailers to make a larger profit, thus better maintaining the growth rate of the expansion of the power retailers

### 4.4. Operation of Energy Conservation Services of Power Retailers Considering Customer-Side Proliferation Stagnation

**Figure 19:**
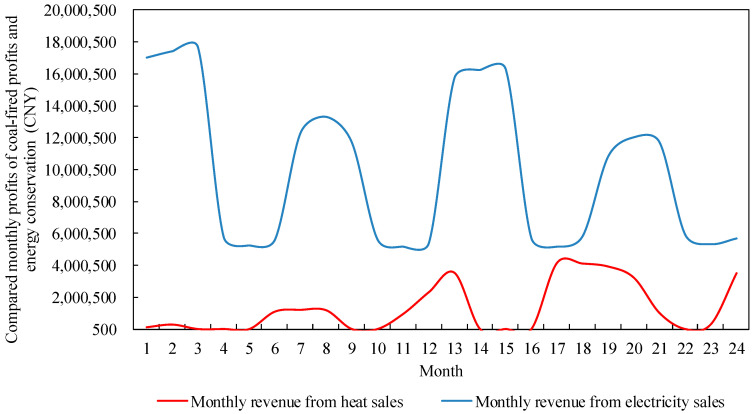
Comparison of monthly electricity sales revenue and heat sales revenue of power retailers.

**Figure 20:**
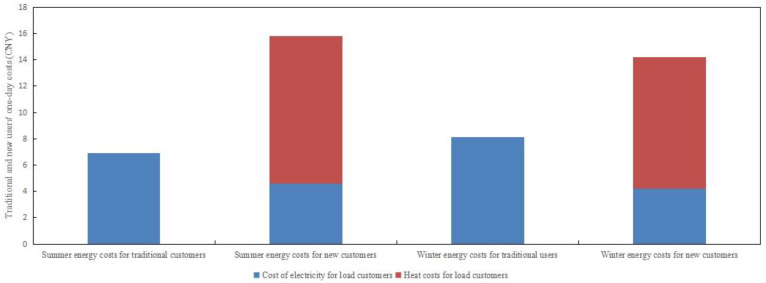
Comparison of typical daily energy cost between traditional users and new users in summer and winter.

The reason for the stagnation of the proliferation of cold-heat-electricity packages on the user side of the load is that coal prices fluctuate greatly in the market and power producers need to purchase a certain percentage of coal as energy consumption and emergency reserves in order to avoid “power outages”, thus increasing the cost of coal-fired power producers. The power retailers set a higher price for heat to ensure its own revenue. During the summer months when customer demand for cooling is high, the cost of conventional air conditioning is lower than the cost of the new package, which in turn leads to a stagnation in customer proliferation. However, combined with the fact that the number of people choosing the new business model for diffusion at the end of the arithmetic simulation in Figure 11 is less than half of the total number of customers in the region, setting a higher heat price will block the diffusion of the cold-heat-electricity multi-energy package on the customer side, and the number of sources of revenue for the power retailers will be suppressed. Additionally, based on the comparison of the revenue from electricity sales with the revenue from heat sales in Figure 19, and the comparison of energy costs for load customers on a typical day in summer and winter in Figure 20, it becomes clearer that the higher heat price brings additional revenue to the power retailers. For the load users who choose the new commercial package, this reduces the use of electricity and, thus, the cost of electricity, but results in a higher cost of heat and cold energy, thus causing a stagnation in the proliferation of the new package on the customer side.

In response, the power retailers have taken the opportunity to introduce an energy guarantee service that no longer uses the high revenue from heat sales as a way to cover the cost hole of rising coal prices. The new cold-heat-electricity multi-energy package has been redefined so that thermal energy is no longer priced for sale, but as an add-on to signing up to the new package. When electricity is used, heat is packaged and sold. The retail tariff, which is 0.2 times higher than the original, is a modest increase relative to the load customers participating in the new business model but provides additional revenue to the power retailers from the sale of electricity.

**Figure 21:**
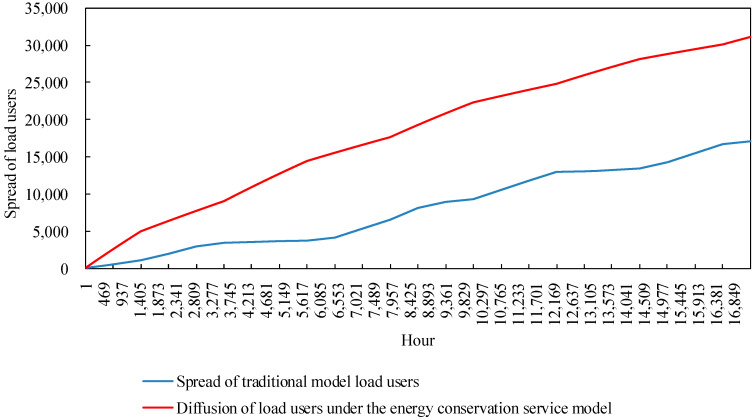
The comparison of the proliferation of load users under energy conservation services.

As a result, the power retailers with a generator background promote to the region’s load customers a cold-heat-electricity multi-energy package that takes into account energy conservation services, namely a slight increase in the retail tariff compared to the normal tariff. In the summer and winter seasons, the free packaged sale of cold and heat energy saves the load users from having to bear significant heat energy costs if they choose this business model, resulting in a significant diffusion rate in the summer and winter seasons. The comparison of load user diffusion is shown in Figure 21. In comparison to the load customer diffusion in the traditional model, the customer-side diffusion avoids the stagnation of diffusion in summer and winter due to the increase in coal prices, while the growth rate increases significantly. At the end of the simulation, the number of load users participating in the cooling-heating-electricity package under energy conservation reached 60% of the total number of users in the region.

**Figure 22:**
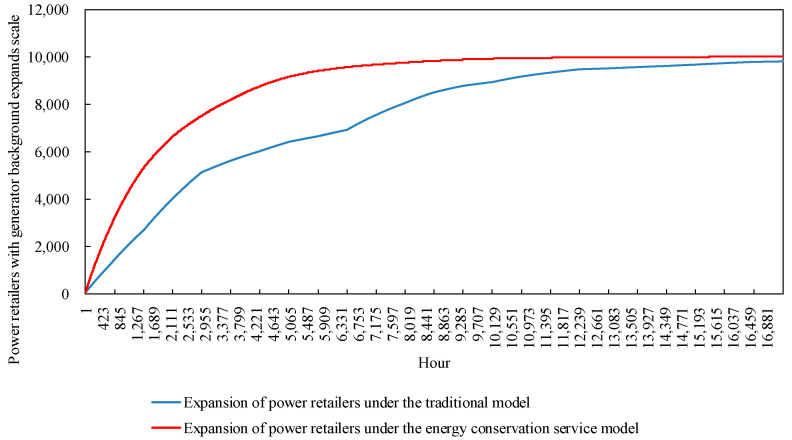
The comparison of the comparative expansion of power retailers under energy conservation services.

At the same time, for the power retailers, although it loses revenue from the sale of heat in the new package considering energy conservation services, it attracts a large number of load customers to participate with a preferential tariff for the sale of hot and cold electricity as a package, which greatly increases the revenue stream of the power retailers. A comparison of the expansion of power retailers considering energy guarantee services is shown in Figure 22. On the one hand, the consideration of energy guarantee services by generators does not affect their own expansion, and, on the other hand, it increases the diffusion of the new package on the load customer side, avoiding the risk of stagnation in the diffusion of customers when promoting the new package.

Most of the retail strategies promoted by traditional power retailers with a generator background are based on their power generation background to buy electricity from their own power generation departments and gain advantages in market bidding [25]. In contrast, the retail strategy of cold-hot-electric multi-energy package considering energy conservation is proposed in this paper. Under the background of the current rising power generation cost and the increasing demand for multi-energy, the improvement is made as shown in Table 9.

## 5. Conclusions

This paper analyzes the expansion of power retailers and the proliferation of load users in the context of smart grid development, based on IOT technology and system dynamics, in the context of a cold-heat-electricity multi-energy package. A model of power retailers with a generator background is developed, and the adoption of energy assurance services by the power retailers to cope with market risks is investigated. The following main conclusions are drawn from the analysis of the case in this paper.

(1) To respond to the construction of the retail market, power retailers with a generator background actively participate in the carbon emissions market and give full play to the advantages of CHP units. This is also based on the IOT’s technical support of data collection, interconnection and calculation for the electricity sales environment, which expands the cold-heat-electricity multi-energy package for load users and improves the efficiency of resource utilization. On the one hand, the multi-energy needs of load users are met, and on the other hand, new sources of income are opened up for the power retailers.
(2) Considering the analysis of the data collection of the market environment of the power retailers, based on the operating characteristics of coal-fired generating units, FGD systems and other equipment, as well as the size of the market, a revenue and cost model regarding the power retailers with a generator background is created. The participation of the power retailers and load users in the operation is simulated to provide the demand for the energy conservation services to be set up by the power retailers.
(3) Through IOT technology to monitor the cost and revenue of the power retailers in response to the risk of rising heat prices due to coal price market fluctuations in the retail market, the power retailers have set up an energy guarantee service. The proportion of energy losses and emergency reserves of the power producer is reduced, and in times of rising coal prices, the power producer’s own coal purchase costs are reduced by purchasing thermal energy from energy assurance service providers to supply customers. The comparative analysis proves that considering energy assurance services can effectively control the cost of power retailers and make profits to facilitate the expansion of power retailers. In the middle of the simulation, the scale of the power retailers increases by 13.103%.
(4) In response to the risk of stagnation of customer-side diffusion in the retail market by power retailers with a generator background, the energy guarantee service was installed. Regarding the new package, proceeds from the sale of heat are forgone and heat and cold energy are sold as a bonus to electricity at an increased tariff. This significantly enhances the diffusion benefits of the new packages on the customer side. Based on real-time monitoring of data by the IOT, a comparative analysis of load customer-side diffusion and the expansion of power retailers is carried out. Considering that the retailing strategy of cooling-heating-electricity multi-energy packages for energy conservation services can effectively enhance the number of customer-side diffusion is proven: its growth trend increases from 1.0225 to 1.7408, with obvious effect, the continuous expansion of the number of users can stabilize the energy conservation security capacity of the power retailers and improve the scale expansion of the power retailers in the region.
(5) Subsequently, the green generation resources that different types of generators have can be considered, and more business models can continue to be studied in depth in terms of power retailers with a generator background, providing more business models for load users.

## Figures and Tables

**Table 1:** Mathematical model for the diffusion of cold-heat-electric multi-energy packages.

Parameters	Formula
Heat consumption per hour for load users	= New customer base heat consumption × Number of new users + Traditional customer base heat consumption × Number of traditional users
Electricity consumption per hour for load users	= New customer base electricity consumption × Number of new users + Traditional customer base electricity consumption × Number of traditional users
Actual coal burned by generators	= min {max {The heat required to set electricity by heat, The heat required to set heat by electricity}/Heat production per unit of standard coal, Amount of coal that can be burned by generators}
User appeal	= 1 + (Traditional user expenditure costs − New user expenditure costs) /Traditional user expenditure costs)

**Table 2:** Cost–benefit mathematical model for desulphurization and carbon emissions.

Parameters	Formula
Carbon emissions per hour from power retailers	= 3.668 × Actual amount of coal burned per hour by generators × (1 − Rate of heat loss from incomplete combustion) × Oxidation rate of carbon × Carbon content of the base received from burning coal [19]
Cost per hour of carbon emissions for power retailers	= Average carbon emissions per hour × Average carbon price per hour
Sulphur emissions per hour for power retailers	= 2 × Actual amount of coal burned per hour by generators × Sulphur content of standard coal × Sulphur dioxide conversion × (1 − FGD efficiency) [20]
Cost per hour of FGD for the power retailers	= Average sulfur emission per hour × Unit Sewage Charges + Production costs for FGD systems
Benefits of FGD systems	= Proceeds from the sale of FGD gypsum + FGD electricity tariff subsidy

**Table 3:** Mathematical model for energy conservation services.

Parameters	Formula
Cost per hour for energy assurance services for power retailers	= Energy conservation service electricity tariff × Energy conservation services electricity supply + Energy conservation service heat tariff × Energy conservation services heat supply + Unit price per hour for energy conservation services
Energy conservation services power supply	= abs {min {Power retailers ‘hourly outgoing transmission volume − Hourly electricity demand of load consumers, 0}}
Energy conservation services heat supply	= abs {min {Power retailers deliver heat every hour − Heat demand per hour for load users, 0}}

**Table 4:** Parameters related to coal-fired units.

Parameters	Data
Heat generated per unit of standard coal burned (kJ/kg)	29,301
Amount of coal required per unit of electricity generated (kg/(kW·h))	0.3
Heat required per unit of electricity generated (kJ/(kW·h))	8000
Power Generation Commercial Coal Reserve Ratio	0.001
Proportion of electricity used by generators’ plants	0.0475
Heat ratio for generators’ plants	0.01
Percentage of operation and maintenance costs for generators	0.323
Unit operation and maintenance costs of energy storage plants/(CNY/(kW·h))	0.6
Hot water storage tank unit operation and maintenance into/(CNY/kJ)	0.00002
Transmission heat loss rate	0.1
Transmission power loss rate	0.0469
Market size	10,000

**Table 5:** Parameters related to FGD systems.

Parameters	Data
Sulphur content of standard coal	0.008
Sulphur dioxide conversion	0.9
Desulphurization gypsum to sulfur dioxide ratio	2.69
Purity of desulphurization gypsum	0.9

**Table 6:** Parameters related to carbon emissions market.

Parameters	Data
Oxidation rate of carbon	0.008
Rate of heat loss from incomplete combustion	0.9
Carbon content of the base received from burning coal	
Carbon price floor	2.69
Carbon price cap	0.9

**Table 7:** Parameters related to load users.

Parameters	Data
Fluctuations in electricity consumption	0.01
Fluctuations in heat consumption	0.02
Average power of user equipment	0.4097
User willingness factor	0.091
Total number of users in the region	50,000

**Table 8:** The related parameters of energy conservation service in power retailers.

Parameters	Data	
Unit Price for Energy Conservation Services (CYN/h)	200	
Energy Conservation Services Heat Tariff (CYN/kJ)	0.000003	
Energy Conservation Service electricity tariff (CYN/(kW·h))	0.25	0 < t < 6 or 22 < t ≤ 24
	0.28	6 ≤ t ≤ 22

**Table 9:** Comparison between the traditional model and the proposed model.

Traditional Model	Proposed Model
Revenue at a lower wholesale price	Revenue is obtained by “fixing electricity by heat” and “fixing heat by electricity” based on multi-energy needs of users
Increased cost burden due to upward fluctuations in coal prices	The proportion of energy loss and emergency reserve of power producers is reduced. During the upward period of coal price, electricity and heat energy are purchased from energy supply service providers for users, thereby reducing the cost of coal purchase.
The diffusion of commercial packages restrained by higher energy supply prices on the user side	By setting up the energy conservation service, abandoning the heat income in the new package to increase the electricity price and packaging the heat and cold energy as a gift for electricity, greatly enhancing the diffusion efficiency of the new package on the user side

## Data Availability

Not applicable.
